# Complete genome sequence of the lignin-degrading bacterium *Klebsiella* sp. strain BRL6-2

**DOI:** 10.1186/1944-3277-9-19

**Published:** 2014-12-08

**Authors:** Hannah L Woo, Nicholas R Ballor, Terry C Hazen, Julian L Fortney, Blake Simmons, Karen Walston Davenport, Lynne Goodwin, Natalia Ivanova, Nikos C Kyrpides, Konstantinos Mavromatis, Tanja Woyke, Janet Jansson, Jeff Kimbrel, Kristen M DeAngelis

**Affiliations:** 1 Microbial Communities Group, Deconstruction Division, Joint BioEnergy Institute, Emeryville, CA, USA; 2 Physical Biosciences Division, Lawrence Berkeley National Laboratory, Berkeley, CA, USA; 3 Department of Civil & Environmental Engineering, The University of Tennessee, Knoxville, TN, USA; 4 Department of Microbiology, The University of Tennessee, Knoxville, TN, USA; 5 Department of Earth & Planetary Sciences, The University of Tennessee, Knoxville, TN, USA; 6 Sandia National Lab, Livermore, CA, USA; 7 Los Alamos National Laboratory, Los Alamos, NM, USA; 8 Department of Energy Joint Genome Institute, Walnut Creek, CA, USA; 9 Biological Sciences Division, Pacific Northwest National Laboratory, Richland, WA, USA; 10 Lawrence Berkeley National Laboratory, Berkeley, CA, USA; 11 Microbiology Department, University of Massachusetts, Amherst, MA, USA

**Keywords:** Anaerobic lignin degradation, Tropical forest soil isolate, Facultative anaerobe

## Abstract

In an effort to discover anaerobic bacteria capable of lignin degradation, we isolated *Klebsiella* sp. strain BRL6-2 on minimal media with alkali lignin as the sole carbon source. This organism was isolated anaerobically from tropical forest soils collected from the Bisley watershed at the Ridge site in the El Yunque National Forest in Puerto Rico, USA, part of the Luquillo Long-Term Ecological Research Station. At this site, the soils experience strong fluctuations in redox potential and are characterized by cycles of iron oxidation and reduction. Genome sequencing was targeted because of its ability to grow on lignin anaerobically and lignocellulolytic activity via *in vitro* enzyme assays. The genome of *Klebsiella* sp. strain BRL6-2 is 5.80 Mbp with no detected plasmids, and includes a relatively small arsenal of genes encoding lignocellulolytic carbohydrate active enzymes. The genome revealed four putative peroxidases including glutathione and DyP-type peroxidases, and a complete protocatechuate pathway encoded in a single gene cluster. Physiological studies revealed *Klebsiella* sp. strain BRL6-2 to be relatively stress tolerant to high ionic strength conditions. It grows in increasing concentrations of ionic liquid (1-ethyl-3-methyl-imidazolium acetate) up to 73.44 mM and NaCl up to 1.5 M.

## Introduction

Lignin is one of the biggest barriers to efficient lignocellulose deconstruction because it occludes the action of cellulases. It is also a major waste stream after lignocellulose deconstruction. Tropical forest soils are the sites of very high rates of decomposition, accompanied by very low and fluctuating redox potential conditions [1,2]. Because early stage decomposition is typically dominated by fungi and the free-radical generating oxidative enzymes phenol oxidase and peroxidase [3,4], we targeted anaerobic tropical forest soils with the idea that they would be dominated by bacterial rather than fungal decomposers. Bacteria grow faster than fungi, allowing higher recombinant enzyme production for commercial use [5]. To discover organisms that were capable of breaking down lignin without the use of oxygen free radicals, we isolated *Klebsiella* sp. strain BRL6-2 under anaerobic conditions using lignin as the sole carbon source. In addition, this strain was observed to withstand moderately high concentrations of ionic liquids, and thus was targeted for whole genome sequencing.

### Organism information

*Klebsiella* sp. strain BRL6-2 was isolated from soil collected from the Bisley watershed at the Ridge site in the El Yunque experimental forest, part of the Luquillo Long-Term Ecological Research Station in Luquillo, Puerto Rico, USA. A soil slurry was made with 1 gram of soil sample diluted in 100 ml of MOD CCMA media without carbon source, serially diluted and inoculated to roll tubes containing MOD CCMA media with alkali lignin as the C source. MOD CCMA media consists of 2.8 g L^-1^ NaCl, 0.1 g L^-1^ KCl, 27 mM MgCl_2_, 1 mM CaCl_2_, 1.25 mM NH_4_Cl, 9.76 g L^-1^ MES, 1.1 ml L^-1^ filter sterilized 1 M K_2_HPO_4_, 12.5 ml L^-1^ trace minerals [6,7], and 1 ml L^-1^ Thauer’s vitamins [8]. Tubes were incubated at room temperature for up to 12 weeks, at which point the colony was picked from a roll tube that had been inoculated with a 10^-4^ dilution of soil slurry, grown in 10% tryptic soy broth (TSB), and characterized.

**Figure 1:**
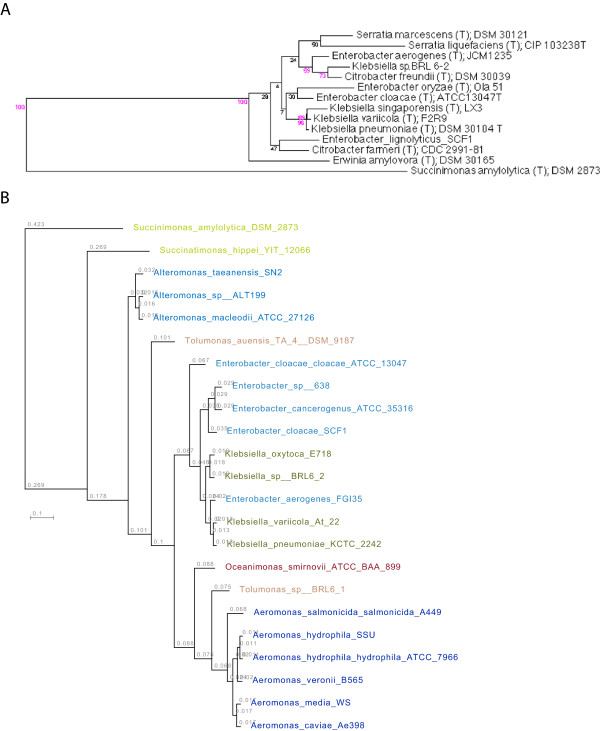
**Phylogenetic trees highlighting the position of *Klebsiella *sp. strain strain BRL6-2 relative to other type and non-type strains within the Gammaproteobacteria, based on (A) 16S ribosomal RNA phylogeny, and (B) whole genome classification based on pfams.** Strains are shown with corresponding NCBI genome project ids listed within [11]. The 16S tree uses sequences aligned by the RDP aligner, the Jukes-Cantor corrected distance model to construct a distance matrix based on alignment model positions without the use of alignment inserts, and a minimum comparable position of 200. The tree is built with RDP Tree Builder, which uses Weighbor [12] with an alphabet size of 4 and length size of 1000. The building of the tree also involves a bootstrapping process repeated 100 times to generate a majority consensus tree [13]. The whole genome classification is a hierarchical clustering of pfams groups that was generated using the Integrated Microbial Genomes (IMG) system [14]. *Succinimonas amylolytica* DSM2873 , *Succinatimonas hippei* YIT12066, and *Tolumonas auensis* TA 4 DSM9187 are type strains with genomes available in IMG. All others are non-type strains.

For initial genotyping and for validating the isolation, the small subunit ribosomal RNA gene was sequenced by Sanger sequencing using the universal primers 8 F and 1492R [9]. The 16S rRNA gene sequence places *Klebsiella* sp. strain BRL6-2 in the domain Bacteria, phylum Proteobacteria, class Gammaproteobacteria, and order Enterobacterales (Figure 1A). However, small subunit ribosomal RNA (16S rRNA) sequence is not sufficient to clearly define the evolutionary history of this region of the *Gammaproteobacteria*, so we have also constructed a hierarchical clustering of whole genomes based on pfams [10] (Figure 1B). This clustering supports the placement of *Klebsiella* sp. strain BRL6-2 within the order Enterobacterales.

**Table 1:** **Classification and general features of *Klebsiella *** **sp. strain BRL6-2**

**MIGS ID**	**Property**	**Term**	**Evidence code**^**a**^
	Current classification	Domain *Bacteria*	TAS [15]
		Phylum *Proteobacteria*	TAS [16]
		Class *Gammaproteobacteria*	TAS [17]
		Order *Enterobacteriales*	TAS [18]
		Family *Enterobacteriaceae*	TAS [19]
		Genus *Klebsiella*	TAS [20,21]
		Species *Klebsiella sp. s*train BRL6-2	TAS [18,19,22]
	Gram stain	negative	NAS
	Cell shape	rod	IDA
	Motility	motile via flagella	IDA
	Sporulation	non-sporulating	IDA
	Temperature range	Mesophile	IDA
	Optimum temperature	30°C	IDA
	pH range; Optimum	8-10; 8	IDA
	Carbon source	glucose, xylose, others (Table 7)	IDA
MIGS-6	Habitat	Tropical forest soils	TAS [23]
MIGS-6.3	Salinity	Can tolerate up to 9% NaCl, 6% KCl. Growth in 10% trypticase soy broth is improved with 0.125 M NaCl	IDA
MIGS-22	Oxygen	facultative aerobe; grows well under completely oxic and anoxic conditions	IDA
MIGS-15	Biotic relationship	free-living	IDA
MIGS-14	Pathogenicity	no	
MIGS-4	Geographic location	Soil collected from a subtropical lower montane wet forest in the Luquillo Experimental Forest, part of the NSF- sponsored Long-Term Ecological Research program in Puerto Rico	IDA
MIGS-5	Sample collection time	July 2009	IDA
MIGS-4.1 MIGS-4.2	Latitude – Longitude	(18.268 N, 65.760 W)	IDA
MIGS-4.3	Depth	10 cm	IDA
MIGS-4.4	Altitude	375 m	IDA

(a) Evidence codes - IDA: Inferred from Direct Assay; TAS: Traceable Author Statement (i.e., a direct report exists in the literature); NAS: Non-traceable Author Statement (i.e., not directly observed for the living, isolated sample, but based on a generally accepted property for the species, or anecdotal evidence). These evidence codes are from the Gene Ontology project [24].

### Genome sequencing information

#### Genome project history

The genome was selected based on the ability of *Klebsiella* sp. strain BRL6-2 to grown on and degrade lignin anaerobically (Table 1). The genome sequence was completed on 1 February 2013, and presented for public access on April 17, 2014 by Genbank. Finishing was completed at Los Alamos National Laboratory. A summary of the project information is shown in Table 2, which also presents the project information and its association with MIGS version 2.0 compliance [25].

**Table 2:** Project information

**MIGS ID**	**Property**	**Term**
MIGS-31	Finishing quality	Permanent draft
MIGS-28	Libraries used	Illumina Std PE, Illumina CLIP, PacBio
MIGS-29	Sequencing platforms	Illumina HiSeq 2000, PacBio
MIGS-31.2	Fold coverage	Illumina Std PE 765x
		Illumina CLIP PE 626x
		PacBio 57x
MIGS-30	Assemblers	AllpathsLG
MIGS-32	Gene calling method	Prodigal 1.4, GenePRIMP
	Locus tag	G360
	Genbank ID	ARVT00000000
	Genbank Date of Release	April 17, 2014
	GOLD ID	Gi0021863
	BIOPROJECT	PRJNA185290
	Project relevance	Anaerobic lignin, switchgrass decomposition
MIGS-13	Source Material Identifier	DSM 25465

### Growth conditions and DNA preparation

*Klebsiella* sp. strain BRL6-2 grows well aerobically and anaerobically, and was routinely cultivated aerobically in 10% tryptic soy broth (TSB) with shaking at 200 rpm at 30°C. DNA for sequencing was obtained using the Qiagen Genomic-tip kit and following the manufacturer’s instructions for the 500/g size extraction. Three column preparations were necessary to obtain 50 μg of high molecular weight DNA. The quantity and quality of the extraction were checked by gel electrophoresis using JGI standards.

### Genome sequencing and assembly

The draft genome of *Klebsiella* sp. strain BRL6–2 was generated at the DOE Joint genome Institute (JGI) using a hybrid of the Illumina and Pacific Biosciences (PacBio) technologies. An Illumina standard shotgun library and long insert mate pair library was constructed and sequenced using the Illumina HiSeq 2000 platform [26]. All general aspects of library construction and sequencing performed at the JGI can be found at http://www.jgi.doe.gov. All raw Illumina sequence data was passed through DUK, a filtering program developed at JGI, which removes known Illumina sequencing and library preparation artifacts [27]. Filtered Illumina and PacBio reads were assembled using AllpathsLG (PrepareAllpathsInputs: PHRED 64 = 1 PLOIDY = 1 FRAG COVERAGE = 125 JUMP COVERAGE = 25; RunAllpath- sLG: THREADS = 8 RUN = standard pairs TARGETS = standard VAPI WARN ONLY = True OVERWRITE = True) [28]. For the Std PE, 25,559,315 reads were generated as raw data, and 25,511,030 (99.811%) reads were output after quality control. For the CLIP PE, 35,554,143 reads were generated as raw data, and 35,548,398 (100% but really 99.984%) reads were output after quality control. A Pacbio SMRTbellTM library was constructed and sequenced on the PacBio RS platform. 81,950 raw PacBio reads yielded 105,417 adapter trimmed and quality filtered subreads totaling 294.3 Mb. The final draft assembly contains one contig in one scaffold. The total size of the genome is 5.8 Mb, and the final assembly provides an average 1199.1X Illumina coverage and 50.7X PacBio coverage of the genome, respectively.

### Genome annotation

Genes were identified using Prodigal [29] as part of the DOE-JGI annotation pipeline [30] followed by a round of manual curation using the JGI GenePRIMP pipeline [31]. The predicted CDSs were translated and used to search the National Center for Biotechnology Information (NCBI) nonredundant database, UniProt, TIGRFam, Pfam, PRIAM, KEGG, COG, and InterPro databases. These data sources were combined to assert a product description for each predicted protein. Additional gene prediction analysis and manual functional annotation was performed within the Integrated Microbial Genomes (IMG-ER) platform (http://img.jgi.doe.gov/er) [32].

### Genome properties

The genome consists of one 5,801,355 bp circular chromosome with no discernable plasmids, and a GC content of 55.24% (Table 3). Of the 5,495 genes predicted, 5,296 were protein-coding genes, and 199 RNAs; 64 pseudogenes were also identified. The majority of the protein-coding genes (86.3%) were assigned with a putative function while the remaining ones were annotated as hypothetical proteins. The distribution of genes into COGs functional categories is presented in Table 4.

**Table 3:** Genome statistics

**Attribute**	**Value**	**% of Total**^**a,b**^
Genome size (bp)	5,801,355	100.00%
DNA coding region (bp)	5,144,694	88.68%
DNA G + C content (bp)	3,204,653	55.24%
DNA scaffolds	1	
Total genes	5,495	100.00%
Protein-coding genes	5,296	96.38%
RNA genes	199	3.62%
Pseudo genes	64	1.16%
Genes in internal clusters	NA	
Genes with function prediction	4,740	86.26%
Genes assigned to COGs	4,599	83.69%
Genes assigned Pfam domains	4,904	89.24%
Genes with signal peptides	582	10.59%
Genes coding for transmembrane helices	1,330	24.20%
CRISPR repeats	NA	

a) The total is based on either the size of the genome in base pairs or the total number of protein coding genes in the annotated genome.

b) Also includes 54 pseudogenes and 5 other genes.

**Table 4:** Number of genes associated with general COG functional categories

**Code**	**Value**	**% of total**^**a**^	**Description**
J	204	3.92	Translation
A	2	0.04	RNA processing and modification
K	489	9.39	Transcription
L	167	3.21	Replication, recombination and repair
B	0	0	Chromatin structure and dynamics
D	38	0.73	Cell cycle control, mitosis and meiosis
V	61	1.17	Defense mechanisms
T	213	4.09	Signal transduction mechanisms
M	261	5.01	Cell wall/membrane biogenesis
N	128	2.46	Cell motility
U	178	3.42	Intracellular trafficking and secretion
O	149	2.86	Post-translational modification, protein turnover, chaperones
C	306	5.88	Energy production and conversion
G	644	12.37	Carbohydrate transport and metabolism
E	508	9.76	Amino acid transport and metabolism
F	113	2.17	Nucleotide transport and metabolism
H	207	3.98	Coenzyme transport and metabolism
I	133	2.55	Lipid transport and metabolism
P	329	6.32	Inorganic ion transport and metabolism
Q	138	2.65	Secondary metabolites biosynthesis, transport and catabolism
R	533	10.24	General function prediction only
S	405	7.78	Function unknown
-	896	16.31	Not in COGs

a) The total is based on the total number of protein coding genes in the annotated genome.

### *Metabolic characterization using biolog phenotypic microarray*

The Biolog phenotypic microarray was used to test *Klebsiella* sp. strain BRL6-2’s utilization of a variety of carbon, nitrogen, phosphorus, and sulfur sources. Different modifications of the isolation medium, MOD CCMA [33], were used to resuspend cells when inoculating different PM plates (Table 5). The scheme is similar to that used with *D. vulgaris* in S. Borglin *et al.*[34]. Plates were done iteratively to optimize each component before proceeding to the next. For all runs, a cell suspension at 0.1 OD_600_ and Biolog redox Dye Mix G were used to inoculate the plates. All plates were prepared in duplicate, incubated at 30°C, and read every 15 minutes for 4.5 days. PM1 and PM2 (carbon sources) were prepared anaerobically and aerobically to compare respiration. The anaerobic plates were prepared anaerobically in the anaerobic chamber in degassed medium and sealed in gas tight Whirlpak bags before loading into the Omnilog reader.

**Table 5:** Inoculation Fluid used for each PM plate type

**PM#**	**Substrates on Plate**	**Inoculating Fluid**
1	Carbon sources	MOD CCMA
2	Carbon sources	MOD CCMA
3	Nitrogen sources	20 mM Mannose MOD CCMA without NH_4_Cl
6	Nitrogen sources	20 mM Mannose MOD CCMA without NH_4_Cl
7	Nitrogen sources	20 mM Mannose MOD CCMA without NH_4_Cl
8	Nitrogen sources	20 mM Mannose MOD CCMA without NH_4_Cl
4	Phosphorus and Sulfur sources	20 mM Mannose MOD CCMA without KH_2_PO_4_ or vitamins
9	Osmolytes	20 mM Mannose MOD CCMA
10	pH	20 mM Mannose MOD CCMA

### Carbon sources

190 different carbon substrates were tested using phenotypic microarray plates. The list of chemical additives that produced the highest increase in respiration relative to background is presented in Table 6. This was measured by the change in redox dye color. D-mannose was used in subsequent plates because of its convenient powder form compared to the viscous Tween solutions, which are mixtures of polyoxyethylene sorbitan esters of saturated fatty acids (predominantly 12:0, 14:0, and 16:0). They are typically used as a surfactant. Although the strain was isolated on lignin, D-cellobiose was utilized at almost the same rate as simpler carbohydrates glucose and xylose, which could suggest possible high cellulolytic activity as well.

**Table 6:** **Carbon sources most utilized by *Klebsiella *** **sp. strain BRL6-2**

**Chemical Name**	**KEGG**	**Ratio to background**
Tween 20	C11624	3.764
Tween 40	N/A	3.573
D-Mannose	C00159	3.678
D-Ribose	C00121	3.425
D-Fructose	C00095	3.602
D-Trehalose	C01083	3.700
N-Acetyl-D-Glucosamine	C03000	3.501
D-Xylose	C00181	3.512
Dulcitol	C01697	3.138
a-D-Glucose	C00031	3.473
D-Cellobiose	C00185	3.434
Background		1

### Anaerobic vs. aerobic carbon source utilization

There were no significant differences between the aerobic and anaerobic utilization of the PM carbon sources. There is a vertical shift in the respiration curves, which is due to a difference in the starting OL at t = 0, as seen in negative control well A01.

### Nitrogen, phosphorus, and sulfur sources

380 nitrogen sources were tested using phenotypic microarray plates. The most utilized nitrogen sources are reported in Table 7. Dipeptide amino acids were some of the most utilized sources, but ammonia from the original MOD CCMA was used in subsequent plates to avoid adding any other potential carbon source. Based on similar reasoning, phosphate was used for subsequent plates (Table 8). Within the sulfur wells, there was robust respiration in the negative control background well indicating that the buffer MES in the MOD CCMA media can serve as a possible sulfur source (Table 9). Since none of the other sulfur sources produced respiration significantly higher than background, MES will serve as the sulfur source in following plates.

**Table 7:** **Nitrogen sources most utilized by *Klebsiella *** **sp. strain BRL6-2**

**N source**	**Ratio to Background**
Gly-Asn	2.867
L-Cysteine	2.835
Gly-Gln	2.766
Allantoin	2.758
Urea	2.749
Ala-Arg	2.677
Ala-Gln	2.650
Thr-Arg	2.634
Trp-Ala	2.631

**Table 8:** **Phosphorus sources most utilized by *Klebsiella *** **sp. strain BRL6-2**

**P source**	**Ratio to Background**
Adenosine 2',3'-Cyclic Monophosphate	1.877
O-Phospho-D-Tyrosine	1.732
Thiophosphate	1.736
Tripolyphosphate	1.810
Phosphoenol Pyruvate	1.733
Cytidine 5'-Monophosphate	1.671
Pyrophosphate	1.767
Phosphate	1.757
Thymidine 5'-Monophosphate	1.677
Guanosine 2',3'-Cyclic Monophosphate	1.686
Guanosine 3'-Monophosphate	1.668
Phospho-Glycolic Acid	1.634
Background	1

**Table 9:** **Sulfur sources most utilized by *Klebsiella *** **sp. strain BRL62**

**S source**	**Ratio to Background**
Tetramethylene Sulfone	1.202
Methane Sulfonic Acid	1.122
L-Methionine Sulfoxide	1.139
N-Acetyl-D,L-Methionine	1.091
L-Djenkolic Acid	1.048
L-Methionine Sulfone	1.114
2-Hydroxyethane Sulfonic Acid	1.039
L-Cysteine Sulfinic Acid	1.090
Gly-Met	1.097
L-Methionine	1.072
Taurocholic Acid	1.020
Thiourea	1.019
Taurine	0.995
Glutathione	1.050
D,L-Lipoamide	1.002
Hypotaurine	1.007
Butane Sulfonic Acid	1.012
N-Acetyl-L-Cysteine	1.005
1-Thio-b-D-Glucose	0.966
Background	1
p-Aminobenzene Sulfonic Acid	0.993
L-Cysteine	1.018
Sulfate	0.990

### Osmolyte stress response

*Klebsiella* sp. strain BRL6-2 was tested for respiration in a variety of osmolyte stressors and a range of pH (Table 10), with and without osmoprotectants (Table 11). For these assays, 20 mM D-Mannose MOD CCMA was used to inoculate the osmolyte response assays in Omnilog PM plates 9 and 10. *Klebsiella* sp. strain BRL6-2 is relatively halotolerant as it grew in increasing concentrations of NaCl up to 9%, which 1.5 M. The addition of trehalose, glycerol, octopine, and trimethylamine-N-oxide aided respiration in presence of 6% NaCl. The strain was found to be particular sensitive to sodium benzoate out of all the osmolytes tested. *Klebsiella* sp. strain BRL6-2 was found to respire at faster rates in pH 8–10, with the optimum at pH 8.

**Table 10:** **Osmolyte Stress Response of *Klebsiella *** **sp. strain BRL6-2**

**Assay Name**	***Klebsiella *** **sp. strain BRL6-2 response**
NaCl Tolerance	Respiration up to 9% (1.5 M)
NaCl Tolerance with various osmoprotectants	See next table for normalized area under the curve
Potassium chloride	Respiration up to 6%
Sodium sulfate	Respiration up to 5%
Ethylene glycol	Respiration up to 20%
Sodium formate	Respiration up to 2%
Urea	Respiration up to 7%
Sodium lactate	Longer lag phase with addition of sodium lactate up to 12% but roughly same final yield.
Sodium phosphate	Respiration in 20–200 mM
Sodium benzoate	No respiration
Ammonium sulfate	Respiration in 10–100 mM
Sodium nitrate	Respiration up to 20 mM
Sodium nitrite	Respiration up to 40 mM

**Table 11:** **Osmoprotectants utilized by *Klebsiella *** **sp. strain BRL6-2 in response to NaCl stress**

**Osmoprotectant**	**Ratio to NaCl 6% without osmoprotectant**
NaCl 6%	1
NaCl 6% + KCl	0.968
NaCl 6% + Creatine	1.011
NaCl 6% + N-acethyl L-glutamine	1.031
NaCl 6% + Sarcosine	1.044
NaCl 6% + L-Carnitine	1.052
NaCl 6% + MOPS	1.121
NaCl 6% + Creatinine	1.131
NaCl 6% + gamma-amino-n-butyric acid	1.145
NaCl 6% + B-glutamic acid	1.150
NaCl 6% + Glutathione	1.154
NaCl 6% + L-proline	1.166
NaCl 6% + Trigonelline	1.195
NaCl 6% + Phosphoryl choline	1.204
NaCl 6% + Betaine	1.240
NaCl 6% + Dimethyl sulphonyl proprionate	1.262
NaCl 6% + Choline	1.285
NaCl 6% + Trimethylamine	1.329
NaCl 6% + N-N Dimethyl glycine	1.329
NaCl 6% + Ectoine	1.333
NaCl 6% + Trimethylamine-N-oxide	1.355
NaCl 6% + Octopine	1.365
NaCl 6% + Glycerol	1.371
NaCl 6% + Trehalose	1.371

### Lignocellulose degradation

Because *Klebsiella* sp. strain BRL6-2 was initially isolated based on colony formation on minimal media with lignin supplied as the sole carbon source [35], we examined the genome to search for genes encoding putative proteins that would be associated with lignin degradation. It has a full protocatechuate pathway for processing catechol degradation to β-ketoadipate, as in *Cupriavidus basilensis* OR16 and *Sphingomonas paucimobilis* SYK6 [36,37]. It has six putative peroxidase genes, encoding for glutathione peroxidases, DyP-type peroxidases, and catalases/peroxidases; all are potentially important for lignin degradation [38,39]. It has two putative lactate dehydrogenase genes (EC:1.1.1.28) and two putative catalase genes (EC:1.11.1.6), and no laccase genes. It also has multiple cytochrome oxidase genes suggesting the possible use of lignin as a terminal electron acceptor as was previously observed for a related isolate *Enterobacter lignolyticus* SCF1 [40]. For the degradation of other relevant lignocellulose components like xylan and cellulose, *Klebsiella* sp. strain BRL6-2 has 2 xylanase genes, 6 β xylosidase genes, 12 β-glucosidase genes, and 2 endoglucanase genes.

Upon isolation of the strain on lignin, *Klebsiella* sp. strain BRL6-2’s ability to degrade several lignocellulose analogs *in vitro* was measured. Using a 4-methylumbelliferone based enzyme assay that has been previously used on bacterial isolates [35], cells grown in MOD CCMA plus 20 mM Mannose had high levels of β-glucosidase and xylosidase activity with 80% and 28% of the given substrate being degraded within 45 hours. However, it had low activity of cellobiohydrolase. *Klebsiella* sp. strain BRL6-2 was also tested for CMCase, another important class of cellulase, using a reducing sugar detection assay with 3,5-dinitrosalicylic acid (DNS) reagent and CMC [41]. No activity was detected on CMC. These low activities of cellulases could not be improved by growing cells in MOD CCMA plus 20 mM Mannose supplemented with 0.1% CMC. Although cellulose was a well-utilized substrate from the phenotypic microarray measurements, it may be due to *Klebsiella* sp. strain BRL6-2’s effective β-glucosidase.

### Ionic liquid tolerance

Currently, ionic liquids are being investigated for their application to the bioenergy feedstock pretreatment; one of which is 1-ethyl-3-methyl-imidazolium acetate (Emim-Acetate). *Klebsiella* sp. strain BRL6-2 was tested for growth in 20 mM Mannose MOD CCMA in the presence of 0 mM, 36.72 mM, 73.44 mM, 146.88 mM, 293.75 mM, 587.51 mM Emim-Acetate. A 6% inoculum concentration from a 0.4 OD_600_ cell suspension was used to inoculate each treatment. Biolog Dye Mix G was used to monitor cell respiration during the incubation at 30°C within a Biolog reader. *Klebsiella* sp. strain BRL6-2 could tolerate up to 73.44 mM Emim-Acetate with increased lag phase and decreased final yields with increasing concentrations of Emim-Acetate. This is not as ionic liquid tolerant as *Enterobacter lignolyticus* SCF1, which was isolated in the same screen and showed tolerance of up to 500 mM 1-ethyl-3-methyl-imidazolium chloride [42]. However, *Klebsiella* sp. strain BRL6-2 tolerates ionic liquid concentrations higher than most bacterial strains, including *E. coli*, which were highly sensitive to concentrations as low as 14.69 mM. *Klebsiella* sp. strain BRL6-2 has 1,107 genes classified as protein coding genes connected to transporters, and these transporters are likley the source of resistance to high ionic strenght, as was also observed in *E. lignolyticus* SCF1 [42].

## Conclusion

*Klebsiella* sp. strain BRL6-2 is an “Enterobacterales” in the order Gammaproteobacteria, originally isolated based on its ability to grow on lignin as sole carbon source under anaerobic conditions. Its ability to degrade lignin likely has origins in its full protocatechuate pathway, six putative peroxidase genes, two putative lactate dehydrogenase genes, and two putative catalase genes. It also has multiple cytochrome oxidase genes, suggesting the possibility of dissimilatory as well as assimilatory lignin degradation pathways. We also observed high tolerance of ionic strenght conditions, likely facilitated by its many transporter classified genes. Future experiments with *Klebsiella* sp. strain BRL6-2 should assess its growth kinetics on purified lignin compounds aerobically and anaerobically to determine the extent of its lignin-degrading potential. However, its fast growth, facultative lifestyle, and tolerance to high ionic strength conditions make it an attractive microbial host to bioengineer for industrial lignocellulose degradation and consolidated bioprocessing of biofuels.

## Competing interests

The authors declare that they have no competing interests.

## Authors' contributions

HLW, NRB, and JLF performed the microbiology and molecular biology studies; KFD, LG, NI, KCK, KM, JK, JJ and TW performed the sequencing and annotation; HLW, KMD performed the genomic analysis; HLW, TCH, BAS and KMD wrote the manuscript.
